# Alternative laronidase dose regimen for patients with mucopolysaccharidosis I: a multinational, retrospective, chart review case series

**DOI:** 10.1186/s13023-016-0437-8

**Published:** 2016-04-29

**Authors:** Dafne Dain Gandelman Horovitz, Angelina X. Acosta, Roberto Giugliani, Anna Hlavatá, Katarína Hlavatá, Michel C. Tchan, Anneliese Lopes Barth, Laercio Cardoso, Emília Katiane Embiruçu de Araújo Leão, Ana Carolina Esposito, Sandra Obikawa Kyosen, Carolina Fischinger Moura De Souza, Ana Maria Martins

**Affiliations:** Instituto Nacional de Saude da Mulher, da Criança e do Adolescente Fernandes Figueira – Fiocruz, Rio de Janeiro, Brazil; Departamento de Pediatria, Serviço de Genética Médica, Universidade Federal da Bahia, Salvador, BA Brazil; Medical Genetics Service, Hospital de Clinicas de Alegre, Porto Alegre, Brazil; 2nd Department of Pediatrics, Comenius University Children´s Hospital, Bratislava, Slovakia; Department of Genetic Medicine, Westmead Hospital and Sydney University, Sydney, Australia; Department of Pediatrics, Universidade Federal de Sao Paulo, São Paulo, Brazil

**Keywords:** Enzyme replacement therapy, α-L-iduronidase deficiency, Clinical outcomes, Tolerability

## Abstract

**Background:**

Enzyme replacement therapy (ERT) with laronidase (recombinant human α-L-iduronidase, Aldurazyme®) is indicated for non-neurological signs and symptoms of mucopolysaccharidosis type I (MPS I). The approved laronidase dose regimen is weekly infusions of 0.58mg/kg, however, patients and caregivers may have difficulty complying with the weekly regimen. We examined clinical outcomes, tolerability, compliance, and satisfaction in a series of patients who switched to every other week infusions.

**Methods:**

This multinational, retrospective, chart review case series analyzed data from 20 patients who had undergone ERT with laronidase 0.58mg/kg weekly for more than one year, and who then switched to 1.2mg/kg every other week.

**Results:**

The majority of patients had attenuated MPS I phenotypes (9 with Hurler-Scheie and 8 with Scheie syndromes) and 3 patients had severe MPS I (Hurler syndrome). Most patients presented with organomegaly (17/20), umbilical and/or inguinal hernia (16/20), cardiac abnormalities (17/20), musculoskeletal abnormalities (19/20), and neurological and/or developmental deficits (15/20). Following laronidase treatment, signs stabilized or improved. No deterioration or reversal of clinical outcome was noted in any patient who switched from the weekly dose of 0.58mg.kg to 1.2mg/kg every other week. There were no safety issues during the duration of every other week dosing. Patient compliance and satisfaction with the dosing regimen were greater with every other week dosing than weekly dosing.

**Conclusions:**

An alternative dose regimen of 1.2mg/kg laronidase every other week was well tolerated and clinically similar to the standard dose for patients who were stabilized with weekly 0.58 mg/kg for one year or more. When an individualized approach to laronidase therapy is necessary, every other week dosing may be an alternative for patients with difficulty receiving weekly infusions.

## Background

Mucopolysaccharidosis type I (MPS I) is an autosomal recessive lysosomal storage disorder caused by α-L-iduronidase (IDUA) deficiency that results in accumulation of the glycosaminoglycans (GAGs) heparan and dermatan sulfate [1]. Accumulation of IDUA substrates causes progressive multisystem disease manifestations, and if left untreated, significant disease burden, disability, and premature death. MPS I has variable phenotypes that range from attenuated to severe classified according to age of symptom onset, rate of disease progression, and the extent of neurological involvement [1, 2]. Patients with Hurler syndrome have the most severe phenotype with onset in infancy and progressive neurocognitive decline. Patients with attenuated phenotypes have either Hurler-Scheie syndrome characterized by onset in early childhood with little to moderate neurocognitive impairment, or Scheie syndrome with onset in childhood and no neurocognitive involvement. Clinical presentations include stiff joints, coarse facial features, hepatomegaly, corneal clouding, hernia, airway-related and neurologic signs and symptoms, and progressive cardiac disease [2, 3]. Incidence of MPS is estimated at 1 per 100,000 live births [4].

Hematopoietic stem cell transplantation (HSCT) is recommended for patients with severe disease before 2 years of age in order to protect the nervous system and prolong survival. Enzyme replacement therapy with laronidase (recombinant human α-L-iduronidase, Aldurazyme®) targets non-neurological manifestations as the recombinant enzyme does not cross the blood-brain barrier. Laronidase reduces biochemical measures of MPS I (urinary GAG excretion) and organ GAG accumulation, and improves functional capacity (e.g., pulmonary function, distance walked, and mobility) [5–7].

The current approved laronidase dose regimen is 0.58 mg/kg (100 U/kg) weekly, administered intravenously for life. Patients and caregivers of patients with MPS I frequently relay to physicians the difficulties of complying with weekly infusion regimen [8]. As a result, physicians may try other dosing regimens for patients in order to improve compliance with therapy. A 26-week dose optimization trial assessed alternative laronidase dose regimens including 1.2 mg/kg dose every other week [9]. There were no significant differences in the reduction of urinary GAG excretion or liver volumes in patients on the weekly or every other week regimens. While incidence of drug-related adverse events was lowest for patients in the weekly regimen, the every other week dose regimen was well tolerated. Therefore, the 1.2 mg/kg every other week regimen may be an alternative for patients with difficulty receiving weekly infusions.

In the absence of long-term studies to assess an every other week laronidase dosing regimen, we examined clinical outcomes, compliance and satisfaction with the dosing regimens in a series of patients who switched to 1.2 mg/kg every other week infusions because of difficulty complying with a weekly infusion schedule.

## Methods

This multinational, retrospective, chart review case series was conducted at the authors’ institutions. All patients with MPS I were diagnosed by biochemical determination of enzyme activity and/or genetic analysis. No selection was made among eligible patients. All patients who had undergone ERT with the recommended laronidase dose of 0.58 mg/kg weekly for more than one year, and who then switched to 1.2 mg/kg every other week were eligible for inclusion. Reasons for changing dosing regimens were recorded in patient records. All patients provided informed consent, which included consent for subsequent data extraction from chart review as needed.

### Data collection

Treating physicians abstracted data on the signs and symptoms of patients with MPS I. Data were collected at diagnosis (Time point 1, T1), at a time point prior to initiating every other week dosing (Time point 2, T2), and following at least 1 year of every 2 week dosing (Time point 3, T3). Data from medical histories and neurological, cardiovascular, pulmonary, gastroenterology, musculoskeletal, and corneal clouding examinations were abstracted and summarized including: duration of ERT, reasons for regimen change, general appearance observed during physical examination, GAG levels and laboratory values, auditory signs, presence of corneal clouding and hernia, results of pulmonary function tests (PFT), liver and spleen size determined by palpation and/or imaging studies and severity based on size, cardiac disease description/severity determined from echocardiogram, joint and skeletal disease observed during physical examination and/or radiographic imaging, presence/severity of dysostosis multiplex, and neurologic and motor development delays assessed during physical examination.

Data on patient compliance with therapy (number of doses received/number of expected doses x 100) and assessment by patients/caregivers of their satisfaction with the change in dosing regimen were summarized.

Incidence of infusion associated reactions (IARs) and any safety issues during every other week dosing were reported.

## Results and discussion

Twenty (20) patients were included in the case analysis. Three patients were diagnosed with severe (Hurler syndrome) and 17 had attenuated (9 with Hurler-Scheie and 8 with Scheie syndromes) disease. Individual treatment profiles and summary statistics are shown in Table 1.

**Table 1:** Treatment Profiles for all Patients

			Laronidase Regimen			
			0.58 mg/kg Weekly		1.2 mg/kg Every other week	
Patient ID	MPS I Phenotype	Age at Dx	Age at Start	Duration	Age at Start	Duration
		years				
1	H	2.42	4.25	1.66	6.00	7.75
2	H	1.17	1.83	4.33	6.17	6.33
3	H	1.67	2.08	1.83	3.92	1.00
	*Mean (SD)*	*1.75 (0.63)*	*2.72 (1.33)*	*2.61 (1.49)*	*5.36 (1.25)*	*5.03 (3.56)*
	*Median* *(Range)*	*1.67*	*2.08*	*1.83*	*6.00*	*6.33*
		*(1.17-2.42)*	*(1.83-4.25)*	*(1.66-4.33)*	*(3.92-6.17)*	*(1.00-7.75)*
4	H-S	1.92	2.42	1.58	4.00	2.00
5	H-S	2.50	5.08	1.50	9.92	2.08
6	H-S	5.17	8.33	1.50	13.00	2.92
7	H-S	2.00	21.00	4.00	25.00	9.00
8	H-S	7.25	9.42	2.70	12.33	1.10
9	H-S	5.00	6.75	1.17	7.91	2.00
10	H-S	15.92	17.17	1.58	18.75	6.75
11	H-S	3.25	4.75	1.33	6.08	5.42
12	H-S	7.50	12.08	1.25	13.33	6.42
	*Mean (SD)*	*5.61(4.4)*	*9.67 (6.11)*	*1.85 (0.92)*	*12.26 (6.49)*	*4.19 (2.77)*
	*Median* *(Range)*	*5.0*	*8.33*	*1.5*	*12.33*	*2.92*
		(1.92–15.92)	(2.42–21.0)	(1.33–4.0)	(4.0–25.0)	(1.1–9.0)
13	S	5.00	6.00	2.00	8.00	6.00
14	S	6.00	6.33	3.42	9.75	2.75
15	S	7.00	10.00	3.00	13.00	6.00
16	S	12.00	12.25	3.42	15.67	2.75
17	S	15.92	20.08	1.59	21.67	6.33
18	S	10.00	19.00	5.00	24.00	3.67
19	S	14.00	18.00	5.00	23.00	3.67
20	S	20.00	21.00	4.00	25.00	5.00
	*Mean (SD)*	*11.24 (5.25)*	*14.08 (6.20)*	*3.43 (1.25)*	*17.51 (6.76)*	*4.52 (1.49)*
	*Median* *(Range)*	*11.00*	*15.13*	*3.42*	*18.67*	*4.34*
		*(5.0–20.0)*	*(6.0–21.0)*	*(1.59–5.00)*	*(8.0–25.0)*	*(2.75–6.33)*

Data in italic are the mean and median of the 3 different patient groups

Patient age at diagnosis was lower in patients with Hurler syndrome (median 1.67 years) and greater than 5 years of age for patients with attenuated disease (medians of 5.0 years for patients with Hurler-Scheie, and 11.0 years for patients with Scheie syndrome). The age of patients at the start of weekly laronidase treatment varied by phenotype, with a median of 2.08 years for patients with Hurler syndrome and 15.13 years for patients with Scheie syndrome. The gap between diagnosis and onset of treatment was longest for patients with attenuated disease. However, the time between diagnosis and treatment for some patients reflected that treatment was not commercially available at the time of diagnosis.

These data are consistent with the pattern of symptom onset and diagnosis times typically observed across MPS I phenotypes [10, 11], where patients with more severe disease have earlier onset and are diagnosed at a younger age than patients with attenuated phenotypes. Importantly, the long-term clinical benefits achieved with ERT appear to be largely dependent on early diagnosis and treatment [12–15]. A case study demonstrated the benefits of early treatment in two siblings with MPS I, where the younger sibling developed few signs and symptoms after starting treatment at 5 months of age compared to an older sibling who began laronidase at 5 years of age and had persistent cardiac and musculoskeletal signs and symptoms [14]. Another case series reported similar findings in three siblings treated with laronidase, where the eldest child who began treatment at age 6 after onset of significant signs and symptoms still had significant clinical features of the disease, while siblings who started therapy at earlier ages (2.5 years and 4 months of age) had milder disease courses [13]. A large multinational case series of 20 patients with attenuated MPS I from 9 sibships where younger siblings received ERT prior to the development of significant signs and symptoms reported that cardiac, musculoskeletal, and cognitive signs and symptoms, when absent or mild in younger siblings at ERT initiation, generally did not develop or progress [15]. These findings suggest that early initiation of ERT prior to the onset of signs and symptoms can slow or prevent the development of severe clinical manifestations. Clinical features such as cardiac valve disease, corneal clouding, and skeletal changes may not respond as well to ERT since irreversible pathology is already present by the time symptoms appear.

The duration of weekly laronidase therapy was ≥ 1 year for all patients and ranged from 1.3 to 5 years (median 1.92). The duration of 1.2 mg/kg every other week ranged from 1 to 9 years (median 3.67). Treatment durations by phenotype are shown in Table 1 and were similar across the groups.

### Clinical presentation at diagnosis

The majority of patients (18/19) presented with coarse facial features and corneal clouding at the time of diagnosis (T1). Hearing loss was reported in 8/18 patients. Most patients had organomegaly (13/19), hernia (umbilical and/or inguinal) (14/19), cardiac abnormalities (15/19), musculoskeletal abnormalities (19/19), and neurological and/or developmental deficits (15/20). As would be expected based on the phenotypic categories, signs were most severe in patients with Hurler syndrome and the least severe in patients with Scheie syndrome. There was considerable variability in the presentations of the 9 patients with Hurler-Scheie phenotype, which is not surprising given the broad age range at which the patients were first treated with laronidase, as shown in Table 1 (range 2.4–21 years).

### Clinical outcomes post-laronidase

Patients with coarse facial features, hearing loss, and corneal clouding at the time of diagnosis had persistence of these signs at T2 and T3, although coarse facial features were noted to improve in some patients. In general, signs stabilized (i.e., they did not progress or never developed), or improved with laronidase treatment and switching to dosing every other week did not reverse the clinical outcome observed following weekly dosing. Importantly, no deterioration was noted in any patient who switched from the weekly dose of 0.58 mg/kg to every other week dosing with 1.2 mg/kg. Since discontinuation of weekly laronidase can be associated with rapid deterioration in clinical presentation [16], these results support that administering a double dose of laronidase every other week maintains adequate enzyme blood levels as has been previously suggested by pharmacodynamic evaluation of every other week dosing regimens in patients with MPS I [9]. No patient reverted to weekly laronidase infusions. While the assessment of changes in severity of clinical signs was limited by the retrospective nature of the study and the lack of a formal scoring system, it is important to note that most of the patients were followed by the same clinical group for many years, and therefore bias in assessing outcomes for the individual patients was minimized. In particular, for severity of musculoskeletal and cardiac disease, imaging results before and after initiating laronidase treatment were assessed by the same individual. Therefore, while a standardized scoring system was not used for determining severity, physicians were better able to assess disease progression or lack thereof for a given patient.

Urinary GAG levels decreased following initiation of the standard laronidase regimen in 15/17 patients, and levels were similar with every 2 week dosing. Levels were within normal ranges for 14/17 patients at T3. Urinary GAG levels at T1, T2, and T3 expressed as the fold increase above the reference or within normal limits (N) are shown in Table 2.

**Table 2:** Measurements of urinary GAGs under weekly and biweekly dose regimens

		Fold increase (mg/GAG/mmol creatinine) above reference range; N = within normal limits		
Patient	Phenotype	T1	T2	T3
1	H	5	N	N
2	H	9	N	N
3	H	19	2.3	3.8
5	HS	16	N	N
6	HS	43	N	N
7	HS	NA	2.9	N
8	HS	4.4	N	N
9	HS	9	N	N
10	HS	23	N	N
11	HS	9.6	N	N
12	HS	4.8	1.2	N
14	S	5	1.2	2.9
16	S	7.9	2.4	1.8
17	S	148	1.1	N
18	S	3.7	N	N
19	S	1.7	2.1	1.2
20	S	2.7	N	1.2

Pulmonary function test data were limited for these patients. Three patients had data at both the T2 and T3 assessments. Patient 7 had Hurler-Scheie syndrome and had FVC and FEV1 values at 33 % and 27 % of predicted normal values, respectively, at T2. These values were essentially unchanged at the T3 assessment (38 % and 30 %, respectively). Two patients with Scheie syndrome had T2 values (82 % and 85 % for FVC, and 94 % and 81 % for FEV1) that were unchanged or slightly improved at T3.

Organomegaly improved, and cardiac, musculoskeletal, and neurodevelopmental signs stabilized or progressed consistent with patterns described in clinical trials and the natural history of the disease [5, 17, 18]. The following sections describe these signs for the individual patients.

#### Organomegaly

**Fig. 1:**
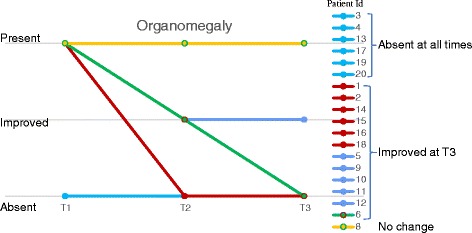
Changes in organomegaly. Organomegaly at time of diagnosis (T1), initiation of laronidase every other week dosing following weekly laronidase dosing regimen (T2), and follow-up assessment (T3). In 6 patients (aqua) there was no organomegaly at any time point. In 6 patients (red), organomegaly present at T1 was absent at T2 and T3. In six of the remaining patients (blue, and green), organomegaly was improved/absent at T3 compared to T1, and in one patient (yellow) organomegaly was not changed at T2 or T3 compared to T1

In general, organomegaly improved at T2 compared to T1 and remained stable at T3, and was absent at T3 in 18/20 patients. In patients with Hurler syndrome, signs improved in 2 patients (Patients 1 and 2) and organomegaly did not develop in the remaining patient (Patient 3). Organomegaly data are summarized in Fig. 1. These results indicate that, similar to the urinary GAG data, most of the patients had already experienced the clinical benefits of undergoing ERT, and that switching to the alternative dose regimen did not compromise the positive outcomes.

#### Cardiac signs

Laronidase is believed to stabilize but not improve cardiac disease when pathological changes are already present at the start of treatment, and early initiation of ERT may help prevent the onset of disease features [13, 19]. As previously reported for patients on long-term laronidase treatment [19], there was no progression to heart failure or cor pulmonale for the patients in this case series, but valve disease did progress in some patients. Patients in this series had a wide range of ages at the time of laronidase initiation as shown in Table 1, with earliest onset of treatment for patients with Hurler syndrome. Patients with Hurler and Hurler-Scheie syndrome had cardiac signs at diagnosis ranging from mitral and/or aortic valve thickening, mitral insufficiency, mitral stenosis, mitral/and or tricuspid valve regurgitation, and cardiomyopathy.

**Fig. 2:**
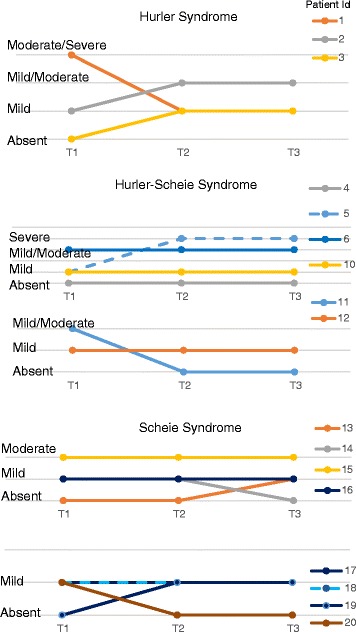
Changes in cardiac valve disease over time for patients with severe (Hurler, panel A) or attenuated MPS I (Hurler-Scheie, panel B and Scheie, panel C). Cardiac symptoms improved in Patients 1, 11, 14 and 20 at T2 or T3. Signs at T1 remained stable at T2 and T3 in Patients 4, 6, 10, 12, 15, 16, 17, and 18. Signs progressed in Patients 2, 3, 5, 13 and 17. Signs at T3 were absent or mild in Patients 1, 4, 10-14, and 16-20

Cardiac signs were mild or absent at all assessments in patients with Scheie syndrome, with the exception of moderate mitral regurgitation in Patient 15 which was stable at T2 and T3. Figure 2 shows the severity of valve disease over time for patients with severity data at all three time points stratified by phenotype, [Hurler Syndrome (A), Hurler-Scheie Syndrome (B) and Scheie Syndrome (C)]. Signs progressed in 4 patients following laronidase treatment (T1 to T2 and/or T3), and were stable in 8 patients. Although improvements were seen in 5 patients, it is generally accepted that valve disease does not benefit from ERT, and perceived improvements may be attributable to the subjective nature of the grading system used in this study and the fact that echocardiograms may have been assessed by different physicians during follow up. Ten of 20 patients had no cardiomyopathy at T1 and cardiomyopathy did not develop following either weekly or every other week laronidase treatment. Five patients had cardiomyopathy at T1 that remained, and 5 patients had cardiomyopathy at T1 but not at the post-treatment assessments.

In summary, cardiac disease type and severity were similar for the weekly and every other week dosing regimen. Despite progression of valve disease in some patients, most patients remained in the mild range for valve disease a T3.

#### Musculoskeletal signs

Improved mobility, endurance, and range of motion are noted in patients following laronidase treatment [7, 12, 17, 19]. However, joint disease and skeletal disease do not generally improve with laronidase treatment if pathological changes are already established [12]. There were sparse data to be able to compare endurance and mobility (e.g., using 6 min walk tests, 6MWT) at diagnosis and at the T2 and T3 assessments. Five patients (#14, 16, 18, 19 and 20) with Scheie syndrome had 6MWT data at all assessments (n = 3) or only the T2 and T3 (n = 2). In all cases, distances walked at the T3 assessment were similar to or greater than distances walked at the T1 and/or T2 assessments, indicating no deterioration in mobility or endurance after commencing every other week dosing with 1.2 mg/kg laronidase.

In general, most patients had dysostosis multiplex and joint contractures of the upper and lower extremities at diagnosis, and these signs persisted at the T2 and T3 assessments. Eleven patients with these signs had no severity data (Patients 4, 5, 6, 8, 9, 10, 11, 13, 15, and 17). For patients with information on the severity of musculoskeletal signs, most had similar impairment at all three time points, which ranged from mild to severe, and was most severe in patients with Hurler syndrome. Among patients with Hurler-Scheie syndrome, musculoskeletal signs ranged from mild to severe and were similar at all assessments.

Patients with Scheie syndrome had milder musculoskeletal impairment than patients with Hurler-Scheie syndrome, and the severity of signs was similar at all assessments, or was similar at T2 and T3 if signs progressed from T1.

#### Neurological and developmental

The severity of neurological or developmental delays at T2 and T3, in general, were similar to the presentations at diagnosis. Among patients with Hurler syndrome, Patient 1 had cognitive delays at all assessments (no data on motor skills). Patient 2 had mild to moderate delays in fine and gross motor skills and delayed cognitive function at all time points, and Patient 3 had severely delayed neurocognitive functions and fine and gross motor skills at T1, with some improvement noted at T3.

As with cardiac and musculoskeletal signs, there was variability in cognitive presentation among patients with Hurler-Scheie syndrome. For patients 4, 5, 9, 10, 11, 6, 7, 8, 9, and 12, sign presentation and severity were similar at all assessments and ranged from normal neurological and developmental assessments to delayed cognitive function with mild to moderate motor development delays.

Most patients with Scheie syndrome had normal cognitive development (Patients 13, 15, and 17, 18, and 19) and normal to mildly impaired fine and gross motor skills (Patients 13, 14, 15, 16, 17, 18, 19, and 20) with similar assessments at all time points. Patients 14 and 16 had moderate range of intellect and no sign of organicity at T1 (determined by medical and psychological assessments), while intellect was subnormal with signs of organicity at T3.

### Patient compliance and satisfaction with the treatment regimen

The reasons for regimen changes varied for patients, but included convenience issues (e.g., the commute to the center for weekly infusions was too long) and quality of life issues (fewer school days missed; fewer caregiver work days missed). All patients responded that they were satisfied, very satisfied, or very pleased with the regimen, that it was a better regimen than the every week schedule, that they were much happier with the regimen, or that QoL improved/significantly improved with no worsening of signs.

Compliance data with the weekly or every other week dosing regimen were available for 10 patients (Patients 1, 5, 6, 8, 9, 10, 11 14, 16, and 17), and demonstrated that compliance increased (median increase of 13 %) when patients changed to every other week dosing. The median (range) compliance during weekly dosing was 80 % (67-88 %) and improved to 93 % (79-100 %) during every other week dosing.

### Tolerability

Physicians reported that there were no safety issues and/or no IARs during the duration of the every other week dosing for 19/20 patients. Data were not available for Patient 15.

## Conclusions

An alternative dose regimen of 1.2 mg/kg laronidase infusions every other week in MPS I patients was well tolerated and clinically similar to the standard dose for patients who were stabilized with 0.58 mg/kg every week for one year or more. Every other week dosing also resulted in greater patient compliance and satisfaction. An individualized approach to laronidase dosing may be an efficient method for improving the treatment condition and therapy outcome of patients with MPS I, however, more detailed prospective studies with larger numbers of patients would be useful to confirm that laronidase 1.2 mg/kg/dose every 2 weeks may be used safely in the long term.
